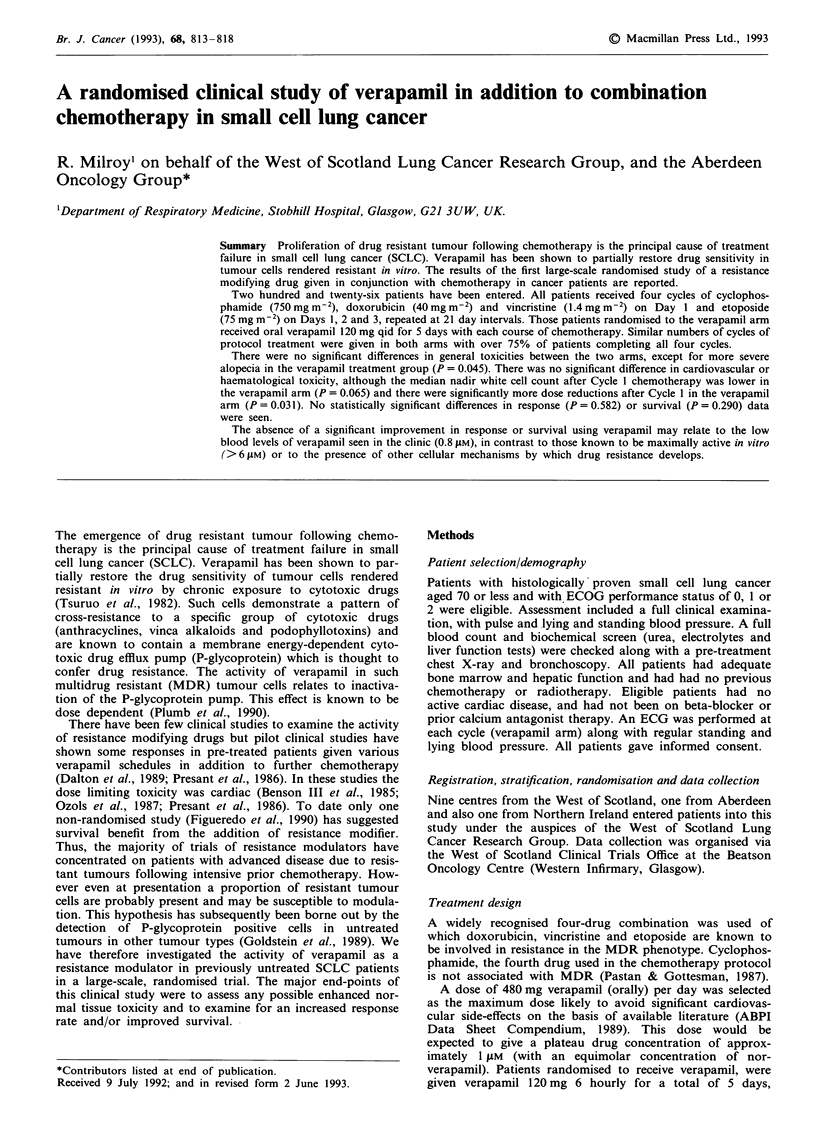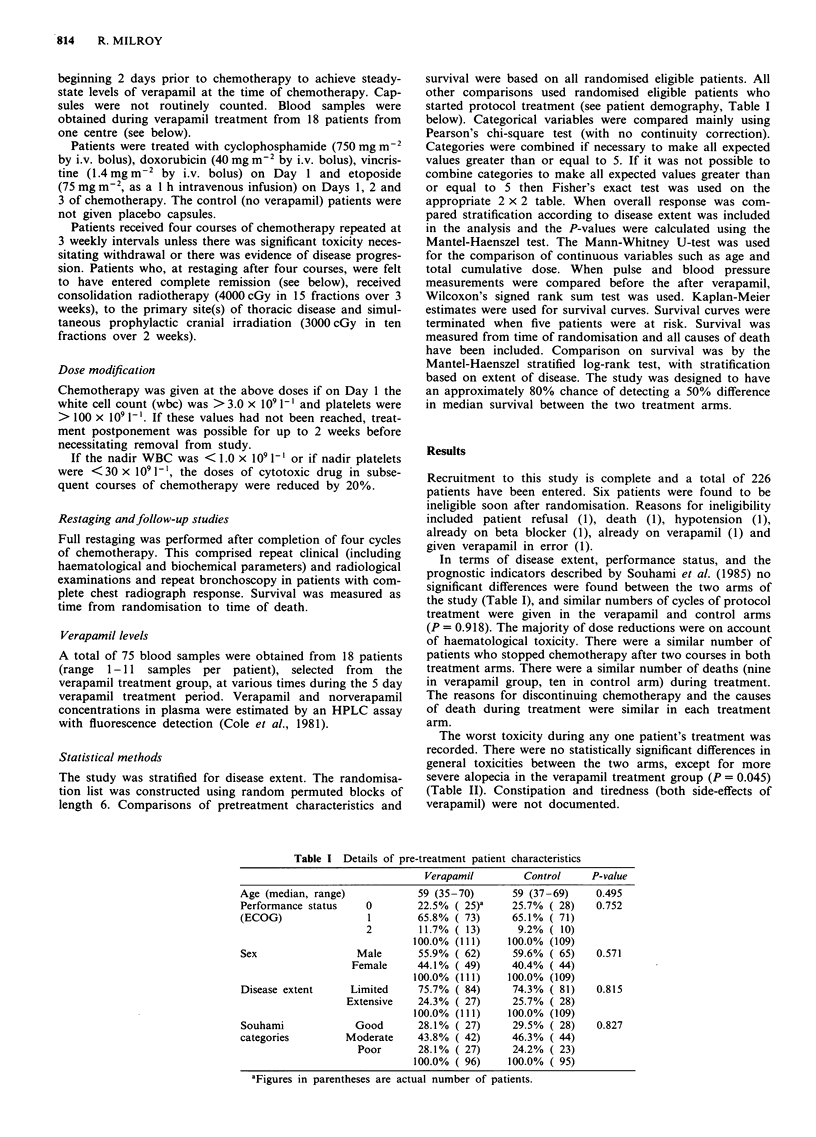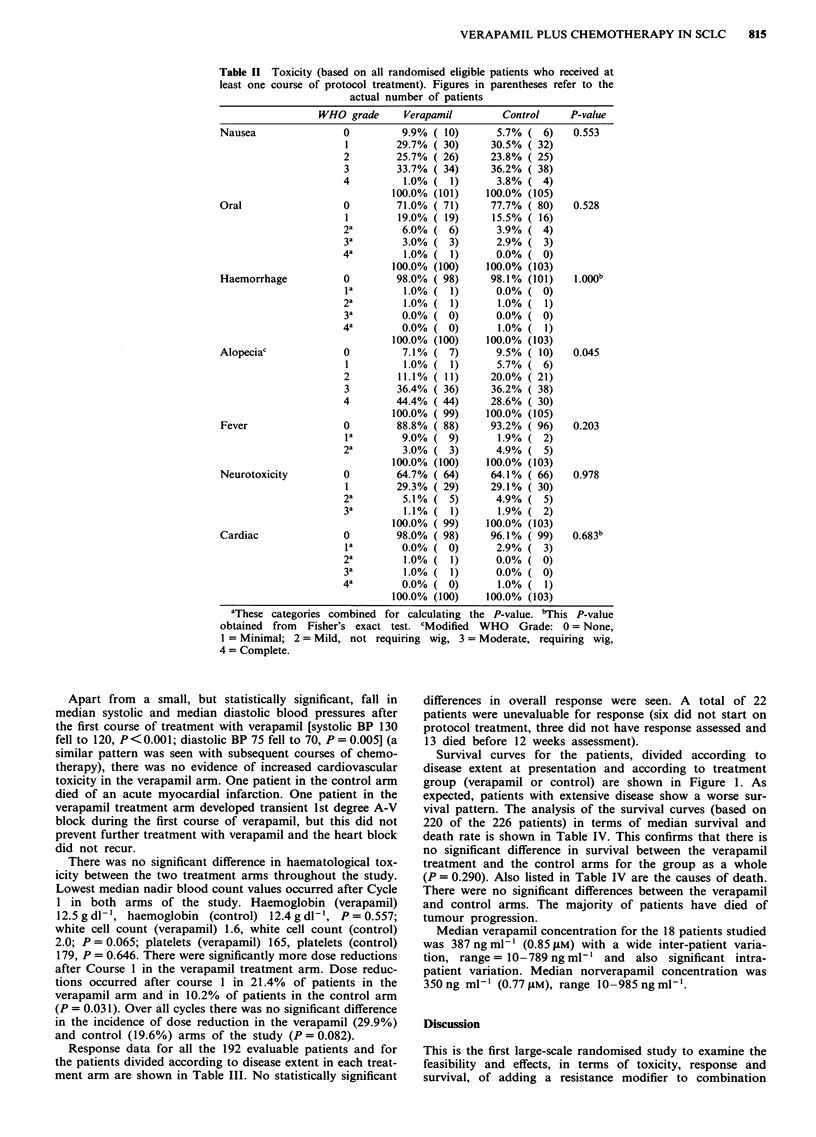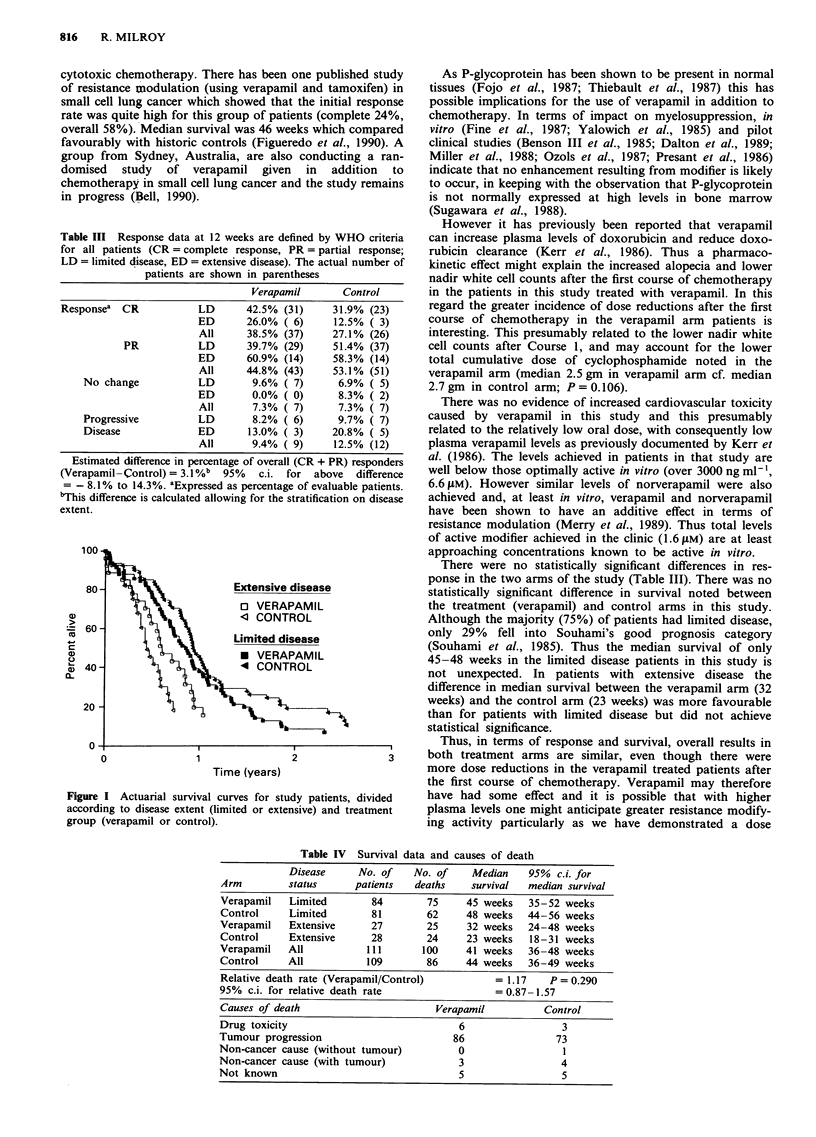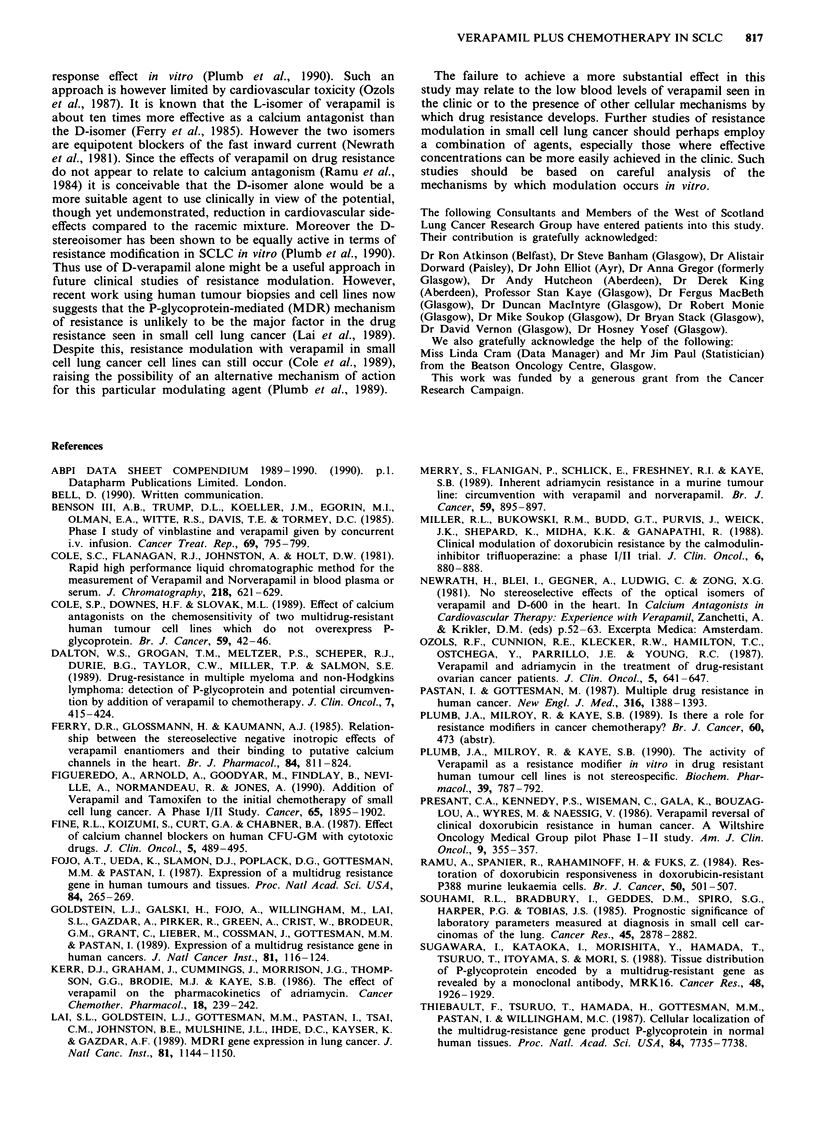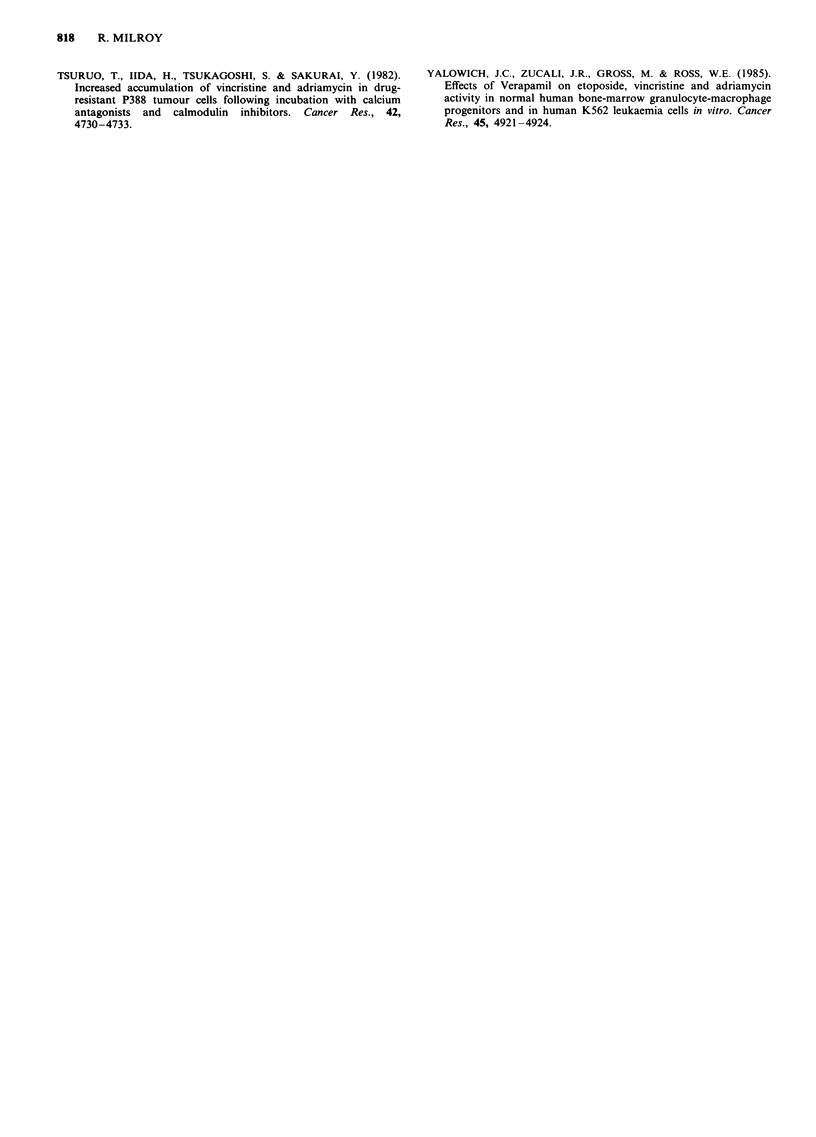# A randomised clinical study of verapamil in addition to combination chemotherapy in small cell lung cancer. West of Scotland Lung Cancer Research Group, and the Aberdeen Oncology Group.

**DOI:** 10.1038/bjc.1993.433

**Published:** 1993-10

**Authors:** R. Milroy

**Affiliations:** Department of Respiratory Medicine, Stobhill Hospital, Glasgow, UK.

## Abstract

Proliferation of drug resistant tumour following chemotherapy is the principal cause of treatment failure in small cell lung cancer (SCLC). Verapamil has been shown to partially restore drug sensitivity in tumour cells rendered resistant in vitro. The results of the first large-scale randomised study of a resistance modifying drug given in conjunction with chemotherapy in cancer patients are reported. Two hundred and twenty-six patients have been entered. All patients received four cycles of cyclophosphamide (750 mg m-2), doxorubicin (40 mg m-2) and vincristine (1.4 mg m-2) on Day 1 and etoposide (75 mg m-2) on Days 1, 2 and 3, repeated at 21 day intervals. Those patients randomised to the verapamil arm received oral verapamil 120 mg qid for 5 days with each course of chemotherapy. Similar numbers of cycles of protocol treatment were given in both arms with over 75% of patients completing all four cycles. There were no significant differences in general toxicities between the two arms, except for more severe alopecia in the verapamil treatment group (P = 0.045). There was no significant difference in cardiovascular or haematological toxicity, although the median nadir white cell count after Cycle 1 chemotherapy was lower in the verapamil arm (P = 0.065) and there were significantly more dose reductions after Cycle 1 in the verapamil arm (P = 0.031). No statistically significant differences in response (P = 0.582) or survival (P = 0.290) data were seen. The absence of a significant improvement in response or survival using verapamil may relate to the low blood levels of verapamil seen in the clinic (0.8 microM), in contrast to those known to be maximally active in vitro (> 6 microM) or to the presence of other cellular mechanisms by which drug resistance develops.


Br. J. Cancer (1993), 68, 813–818 © Macmillan Press Ltd., 1993

### A randomised clinical study of verapamil in addition to combination chemotherapy in small cell lung cancer

R. Milroy[1] on behalf of the West of Scotland Lung Cancer Research Group, and the Aberdeen Oncology Group*

[1]*Department of Respiratory Medicine, Stobhill Hospital, Glasgow, G21 3UW, UK.*

**Summary** Proliferation of drug resistant tumour following chemotherapy is the principal cause of treatment failure in small cell lung cancer (SCLC). Verapamil has been shown to partially restore drug sensitivity in tumour cells rendered resistant *in vitro*. The results of the first large-scale randomised study of a resistance modifying drug given in conjunction with chemotherapy in cancer patients are reported.

Two hundred and twenty-six patients have been entered. All patients received four cycles of cyclophosphamide (750 mg $m^{-2}$), doxorubicin (40 mg $m^{-2}$) and vincristine (1.4 mg $m^{-2}$) on Day 1 and etoposide (75 mg $m^{-2}$) on Days 1, 2 and 3, repeated at 21 day intervals. Those patients randomised to the verapamil arm received oral verapamil 120 mg qid for 5 days with each course of chemotherapy. Similar numbers of cycles of protocol treatment were given in both arms with over 75% of patients completing all four cycles.

There were no significant differences in general toxicities between the two arms, except for more severe alopecia in the verapamil treatment group ($P = 0.045$). There was no significant difference in cardiovascular or haematological toxicity, although the median nadir white cell count after Cycle 1 chemotherapy was lower in the verapamil arm ($P = 0.065$) and there were significantly more dose reductions after Cycle 1 in the verapamil arm ($P = 0.031$). No statistically significant differences in response ($P = 0.582$) or survival ($P = 0.290$) data were seen.

The absence of a significant improvement in response or survival using verapamil may relate to the low blood levels of verapamil seen in the clinic (0.8 μM), in contrast to those known to be maximally active *in vitro* (>6 μM) or to the presence of other cellular mechanisms by which drug resistance develops.

The emergence of drug resistant tumour following chemotherapy is the principal cause of treatment failure in small cell lung cancer (SCLC). Verapamil has been shown to partially restore the drug sensitivity of tumour cells rendered resistant *in vitro* by chronic exposure to cytotoxic drugs (Tsuruo *et al.*, 1982). Such cells demonstrate a pattern of cross-resistance to a specific group of cytotoxic drugs (anthracyclines, vinca alkaloids and podophyllotoxins) and are known to contain a membrane energy-dependent cytotoxic drug efflux pump (P-glycoprotein) which is thought to confer drug resistance. The activity of verapamil in such multidrug resistant (MDR) tumour cells relates to inactivation of the P-glycoprotein pump. This effect is known to be dose dependent (Plumb *et al.*, 1990).

There have been few clinical studies to examine the activity of resistance modifying drugs but pilot clinical studies have shown some responses in pre-treated patients given various verapamil schedules in addition to further chemotherapy (Dalton *et al.*, 1989; Presant *et al.*, 1986). In these studies the dose limiting toxicity was cardiac (Benson III *et al.*, 1985; Ozols *et al.*, 1987; Presant *et al.*, 1986). To date only one non-randomised study (Figueredo *et al.*, 1990) has suggested survival benefit from the addition of resistance modifier. Thus, the majority of trials of resistance modulators have concentrated on patients with advanced disease due to resistant tumours following intensive prior chemotherapy. However even at presentation a proportion of resistant tumour cells are probably present and may be susceptible to modulation. This hypothesis has subsequently been borne out by the detection of P-glycoprotein positive cells in untreated tumours in other tumour types (Goldstein *et al.*, 1989). We have therefore investigated the activity of verapamil as a resistance modulator in previously untreated SCLC patients in a large-scale, randomised trial. The major end-points of this clinical study were to assess any possible enhanced normal tissue toxicity and to examine for an increased response rate and/or improved survival.

*Contributors listed at end of publication.

Received 9 July 1992; and in revised form 2 June 1993.

### Methods

### *Patient selection/demography*

Patients with histologically proven small cell lung cancer aged 70 or less and with ECOG performance status of 0, 1 or 2 were eligible. Assessment included a full clinical examination, with pulse and lying and standing blood pressure. A full blood count and biochemical screen (urea, electrolytes and liver function tests) were checked along with a pre-treatment chest X-ray and bronchoscopy. All patients had adequate bone marrow and hepatic function and had had no previous chemotherapy or radiotherapy. Eligible patients had no active cardiac disease, and had not been on beta-blocker or prior calcium antagonist therapy. An ECG was performed at each cycle (verapamil arm) along with regular standing and lying blood pressure. All patients gave informed consent.

### *Registration, stratification, randomisation and data collection*

Nine centres from the West of Scotland, one from Aberdeen and also one from Northern Ireland entered patients into this study under the auspices of the West of Scotland Lung Cancer Research Group. Data collection was organised via the West of Scotland Clinical Trials Office at the Beatson Oncology Centre (Western Infirmary, Glasgow).

### *Treatment design*

A widely recognised four-drug combination was used of which doxorubicin, vincristine and etoposide are known to be involved in resistance in the MDR phenotype. Cyclophosphamide, the fourth drug used in the chemotherapy protocol is not associated with MDR (Pastan & Gottesman, 1987).

A dose of 480 mg verapamil (orally) per day was selected as the maximum dose likely to avoid significant cardiovascular side-effects on the basis of available literature (ABPI Data Sheet Compendium, 1989). This dose would be expected to give a plateau drug concentration of approximately 1 μM (with an equimolar concentration of norverapamil). Patients randomised to receive verapamil, were given verapamil 120 mg 6 hourly for a total of 5 days, beginning 2 days prior to chemotherapy to achieve steady-state levels of verapamil at the time of chemotherapy. Capsules were not routinely counted. Blood samples were obtained during verapamil treatment from 18 patients from one centre (see below).

Patients were treated with cyclophosphamide (750 mg $m^{-2}$ by i.v. bolus), doxorubicin (40 mg $m^{-2}$ by i.v. bolus), vincristine (1.4 mg $m^{-2}$ by i.v. bolus) on Day 1 and etoposide (75 mg $m^{-2}$, as a 1 h intravenous infusion) on Days 1, 2 and 3 of chemotherapy. The control (no verapamil) patients were not given placebo capsules.

Patients received four courses of chemotherapy repeated at 3 weekly intervals unless there was significant toxicity necessitating withdrawal or there was evidence of disease progression. Patients who, at restaging after four courses, were felt to have entered complete remission (see below), received consolidation radiotherapy (4000 cGy in 15 fractions over 3 weeks), to the primary site(s) of thoracic disease and simultaneous prophylactic cranial irradiation (3000 cGy in ten fractions over 2 weeks).

*Dose modification*

Chemotherapy was given at the above doses if on Day 1 the white cell count (wbc) was $>3.0 \times 10^9\,l^{-1}$ and platelets were $>100 \times 10^9\,l^{-1}$. If these values had not been reached, treatment postponement was possible for up to 2 weeks before necessitating removal from study.

If the nadir WBC was $<1.0 \times 10^9\,l^{-1}$ or if nadir platelets were $<30 \times 10^9\,l^{-1}$, the doses of cytotoxic drug in subsequent courses of chemotherapy were reduced by 20%.

*Restaging and follow-up studies*

Full restaging was performed after completion of four cycles of chemotherapy. This comprised repeat clinical (including haematological and biochemical parameters) and radiological examinations and repeat bronchoscopy in patients with complete chest radiograph response. Survival was measured as time from randomisation to time of death.

*Verapamil levels*

A total of 75 blood samples were obtained from 18 patients (range 1–11 samples per patient), selected from the verapamil treatment group, at various times during the 5 day verapamil treatment period. Verapamil and norverapamil concentrations in plasma were estimated by an HPLC assay with fluorescence detection (Cole *et al.*, 1981).

*Statistical methods*

The study was stratified for disease extent. The randomisation list was constructed using random permuted blocks of length 6. Comparisons of pretreatment characteristics and survival were based on all randomised eligible patients. All other comparisons used randomised eligible patients who started protocol treatment (see patient demography, Table I below). Categorical variables were compared mainly using Pearson's chi-square test (with no continuity correction). Categories were combined if necessary to make all expected values greater than or equal to 5. If it was not possible to combine categories to make all expected values greater than or equal to 5 then Fisher's exact test was used on the appropriate 2 × 2 table. When overall response was compared stratification according to disease extent was included in the analysis and the *P*-values were calculated using the Mantel-Haenszel test. The Mann-Whitney U-test was used for the comparison of continuous variables such as age and total cumulative dose. When pulse and blood pressure measurements were compared before the after verapamil, Wilcoxon's signed rank sum test was used. Kaplan-Meier estimates were used for survival curves. Survival curves were terminated when five patients were at risk. Survival was measured from time of randomisation and all causes of death have been included. Comparison on survival was by the Mantel-Haenszel stratified log-rank test, with stratification based on extent of disease. The study was designed to have an approximately 80% chance of detecting a 50% difference in median survival between the two treatment arms.

### Results

Recruitment to this study is complete and a total of 226 patients have been entered. Six patients were found to be ineligible soon after randomisation. Reasons for ineligibility included patient refusal (1), death (1), hypotension (1), already on beta blocker (1), already on verapamil (1) and given verapamil in error (1).

In terms of disease extent, performance status, and the prognostic indicators described by Souhami *et al.* (1985) no significant differences were found between the two arms of the study (Table I), and similar numbers of cycles of protocol treatment were given in the verapamil and control arms ($P = 0.918$). The majority of dose reductions were on account of haematological toxicity. There were a similar number of patients who stopped chemotherapy after two courses in both treatment arms. There were a similar number of deaths (nine in verapamil group, ten in control arm) during treatment. The reasons for discontinuing chemotherapy and the causes of death during treatment were similar in each treatment arm.

The worst toxicity during any one patient's treatment was recorded. There were no statistically significant differences in general toxicities between the two arms, except for more severe alopecia in the verapamil treatment group ($P = 0.045$) (Table II). Constipation and tiredness (both side-effects of verapamil) were not documented.

**Table I** Details of pre-treatment patient characteristics

| | | *Verapamil* | *Control* | *P-value* |
|---|---|---|---|---|
| Age (median, range) | | 59 (35–70) | 59 (37–69) | 0.495 |
| Performance status | 0 | 22.5% ( 25)[a] | 25.7% ( 28) | 0.752 |
| (ECOG) | 1 | 65.8% ( 73) | 65.1% ( 71) | |
| | 2 | 11.7% ( 13) | 9.2% ( 10) | |
| | | 100.0% (111) | 100.0% (109) | |
| Sex | Male | 55.9% ( 62) | 59.6% ( 65) | 0.571 |
| | Female | 44.1% ( 49) | 40.4% ( 44) | |
| | | 100.0% (111) | 100.0% (109) | |
| Disease extent | Limited | 75.7% ( 84) | 74.3% ( 81) | 0.815 |
| | Extensive | 24.3% ( 27) | 25.7% ( 28) | |
| | | 100.0% (111) | 100.0% (109) | |
| Souhami | Good | 28.1% ( 27) | 29.5% ( 28) | 0.827 |
| categories | Moderate | 43.8% ( 42) | 46.3% ( 44) | |
| | Poor | 28.1% ( 27) | 24.2% ( 23) | |
| | | 100.0% ( 96) | 100.0% ( 95) | |

[a]Figures in parentheses are actual number of patients.

**Table II** Toxicity (based on all randomised eligible patients who received at least one course of protocol treatment). Figures in parentheses refer to the actual number of patients

| | *WHO grade* | *Verapamil* | *Control* | *P-value* |
|---|---|---|---|---|
| Nausea | 0 | 9.9% ( 10) | 5.7% ( 6) | 0.553 |
| | 1 | 29.7% ( 30) | 30.5% ( 32) | |
| | 2 | 25.7% ( 26) | 23.8% ( 25) | |
| | 3 | 33.7% ( 34) | 36.2% ( 38) | |
| | 4 | 1.0% ( 1) | 3.8% ( 4) | |
| | | 100.0% (101) | 100.0% (105) | |
| Oral | 0 | 71.0% ( 71) | 77.7% ( 80) | 0.528 |
| | 1 | 19.0% ( 19) | 15.5% ( 16) | |
| | 2[a] | 6.0% ( 6) | 3.9% ( 4) | |
| | 3[a] | 3.0% ( 3) | 2.9% ( 3) | |
| | 4[a] | 1.0% ( 1) | 0.0% ( 0) | |
| | | 100.0% (100) | 100.0% (103) | |
| Haemorrhage | 0 | 98.0% ( 98) | 98.1% (101) | 1.000[b] |
| | 1[a] | 1.0% ( 1) | 0.0% ( 0) | |
| | 2[a] | 1.0% ( 1) | 1.0% ( 1) | |
| | 3[a] | 0.0% ( 0) | 0.0% ( 0) | |
| | 4[a] | 0.0% ( 0) | 1.0% ( 1) | |
| | | 100.0% (100) | 100.0% (103) | |
| Alopecia[c] | 0 | 7.1% ( 7) | 9.5% ( 10) | 0.045 |
| | 1 | 1.0% ( 1) | 5.7% ( 6) | |
| | 2 | 11.1% ( 11) | 20.0% ( 21) | |
| | 3 | 36.4% ( 36) | 36.2% ( 38) | |
| | 4 | 44.4% ( 44) | 28.6% ( 30) | |
| | | 100.0% ( 99) | 100.0% (105) | |
| Fever | 0 | 88.8% ( 88) | 93.2% ( 96) | 0.203 |
| | 1[a] | 9.0% ( 9) | 1.9% ( 2) | |
| | 2[a] | 3.0% ( 3) | 4.9% ( 5) | |
| | | 100.0% (100) | 100.0% (103) | |
| Neurotoxicity | 0 | 64.7% ( 64) | 64.1% ( 66) | 0.978 |
| | 1 | 29.3% ( 29) | 29.1% ( 30) | |
| | 2[a] | 5.1% ( 5) | 4.9% ( 5) | |
| | 3[a] | 1.1% ( 1) | 1.9% ( 2) | |
| | | 100.0% ( 99) | 100.0% (103) | |
| Cardiac | 0 | 98.0% ( 98) | 96.1% ( 99) | 0.683[b] |
| | 1[a] | 0.0% ( 0) | 2.9% ( 3) | |
| | 2[a] | 1.0% ( 1) | 0.0% ( 0) | |
| | 3[a] | 1.0% ( 1) | 0.0% ( 0) | |
| | 4[a] | 0.0% ( 0) | 1.0% ( 1) | |
| | | 100.0% (100) | 100.0% (103) | |

[a]These categories combined for calculating the *P*-value. [b]This *P*-value obtained from Fisher's exact test. [c]Modified WHO Grade: 0 = None, 1 = Minimal; 2 = Mild, not requiring wig, 3 = Moderate, requiring wig, 4 = Complete.

Apart from a small, but statistically significant, fall in median systolic and median diastolic blood pressures after the first course of treatment with verapamil [systolic BP 130 fell to 120, $P<0.001$; diastolic BP 75 fell to 70, $P=0.005$] (a similar pattern was seen with subsequent courses of chemotherapy), there was no evidence of increased cardiovascular toxicity in the verapamil arm. One patient in the control arm died of an acute myocardial infarction. One patient in the verapamil treatment arm developed transient 1st degree A-V block during the first course of verapamil, but this did not prevent further treatment with verapamil and the heart block did not recur.

There was no significant difference in haematological toxicity between the two treatment arms throughout the study. Lowest median nadir blood count values occurred after Cycle 1 in both arms of the study. Haemoglobin (verapamil) 12.5 g dl$^{-1}$, haemoglobin (control) 12.4 g dl$^{-1}$, $P=0.557$; white cell count (verapamil) 1.6, white cell count (control) 2.0; $P=0.065$; platelets (verapamil) 165, platelets (control) 179, $P=0.646$. There were significantly more dose reductions after Course 1 in the verapamil treatment arm. Dose reductions occurred after course 1 in 21.4% of patients in the verapamil arm and in 10.2% of patients in the control arm ($P=0.031$). Over all cycles there was no significant difference in the incidence of dose reduction in the verapamil (29.9%) and control (19.6%) arms of the study ($P=0.082$).

Response data for all the 192 evaluable patients and for the patients divided according to disease extent in each treatment arm are shown in Table III. No statistically significant differences in overall response were seen. A total of 22 patients were unevaluable for response (six did not start on protocol treatment, three did not have response assessed and 13 died before 12 weeks assessment).

Survival curves for the patients, divided according to disease extent at presentation and according to treatment group (verapamil or control) are shown in Figure 1. As expected, patients with extensive disease show a worse survival pattern. The analysis of the survival curves (based on 220 of the 226 patients) in terms of median survival and death rate is shown in Table IV. This confirms that there is no significant difference in survival between the verapamil treatment and the control arms for the group as a whole ($P=0.290$). Also listed in Table IV are the causes of death. There were no significant differences between the verapamil and control arms. The majority of patients have died of tumour progression.

Median verapamil concentration for the 18 patients studied was 387 ng ml$^{-1}$ (0.85 $\mu$M) with a wide inter-patient variation, range = 10–789 ng ml$^{-1}$ and also significant intra-patient variation. Median norverapamil concentration was 350 ng ml$^{-1}$ (0.77 $\mu$M), range 10–985 ng ml$^{-1}$.

### Discussion

This is the first large-scale randomised study to examine the feasibility and effects, in terms of toxicity, response and survival, of adding a resistance modifier to combination cytotoxic chemotherapy. There has been one published study of resistance modulation (using verapamil and tamoxifen) in small cell lung cancer which showed that the initial response rate was quite high for this group of patients (complete 24%, overall 58%). Median survival was 46 weeks which compared favourably with historic controls (Figueredo *et al.*, 1990). A group from Sydney, Australia, are also conducting a randomised study of verapamil given in addition to chemotherapy in small cell lung cancer and the study remains in progress (Bell, 1990).

**Table III** Response data at 12 weeks are defined by WHO criteria for all patients (CR = complete response, PR = partial response; LD = limited disease, ED = extensive disease). The actual number of patients are shown in parentheses

| | | | *Verapamil* | *Control* |
|---|---|---|---|---|
| Response[a] | CR | LD | 42.5% (31) | 31.9% (23) |
| | | ED | 26.0% ( 6) | 12.5% ( 3) |
| | | All | 38.5% (37) | 27.1% (26) |
| | PR | LD | 39.7% (29) | 51.4% (37) |
| | | ED | 60.9% (14) | 58.3% (14) |
| | | All | 44.8% (43) | 53.1% (51) |
| No change | | LD | 9.6% ( 7) | 6.9% ( 5) |
| | | ED | 0.0% ( 0) | 8.3% ( 2) |
| | | All | 7.3% ( 7) | 7.3% ( 7) |
| Progressive | | LD | 8.2% ( 6) | 9.7% ( 7) |
| Disease | | ED | 13.0% ( 3) | 20.8% ( 5) |
| | | All | 9.4% ( 9) | 12.5% (12) |

Estimated difference in percentage of overall (CR + PR) responders (Verapamil-Control) = 3.1%[b] 95% c.i. for above difference = − 8.1% to 14.3%. [a]Expressed as percentage of evaluable patients. [b]This difference is calculated allowing for the stratification on disease extent.

**Figure 1** Actuarial survival curves for study patients, divided according to disease extent (limited or extensive) and treatment group (verapamil or control).

As P-glycoprotein has been shown to be present in normal tissues (Fojo *et al.*, 1987; Thiebault *et al.*, 1987) this has possible implications for the use of verapamil in addition to chemotherapy. In terms of impact on myelosuppression, *in vitro* (Fine *et al.*, 1987; Yalowich *et al.*, 1985) and pilot clinical studies (Benson III *et al.*, 1985; Dalton *et al.*, 1989; Miller *et al.*, 1988; Ozols *et al.*, 1987; Presant *et al.*, 1986) indicate that no enhancement resulting from modifier is likely to occur, in keeping with the observation that P-glycoprotein is not normally expressed at high levels in bone marrow (Sugawara *et al.*, 1988).

However it has previously been reported that verapamil can increase plasma levels of doxorubicin and reduce doxorubicin clearance (Kerr *et al.*, 1986). Thus a pharmacokinetic effect might explain the increased alopecia and lower nadir white cell counts after the first course of chemotherapy in the patients in this study treated with verapamil. In this regard the greater incidence of dose reductions after the first course of chemotherapy in the verapamil arm patients is interesting. This presumably related to the lower nadir white cell counts after Course 1, and may account for the lower total cumulative dose of cyclophosphamide noted in the verapamil arm (median 2.5 gm in verapamil arm cf. median 2.7 gm in control arm; $P = 0.106$).

There was no evidence of increased cardiovascular toxicity caused by verapamil in this study and this presumably related to the relatively low oral dose, with consequently low plasma verapamil levels as previously documented by Kerr *et al.* (1986). The levels achieved in patients in that study are well below those optimally active *in vitro* (over 3000 ng ml$^{-1}$, 6.6 μM). However similar levels of norverapamil were also achieved and, at least *in vitro*, verapamil and norverapamil have been shown to have an additive effect in terms of resistance modulation (Merry *et al.*, 1989). Thus total levels of active modifier achieved in the clinic (1.6 μM) are at least approaching concentrations known to be active *in vitro*.

There were no statistically significant differences in response in the two arms of the study (Table III). There was no statistically significant difference in survival noted between the treatment (verapamil) and control arms in this study. Although the majority (75%) of patients had limited disease, only 29% fell into Souhami's good prognosis category (Souhami *et al.*, 1985). Thus the median survival of only 45–48 weeks in the limited disease patients in this study is not unexpected. In patients with extensive disease the difference in median survival between the verapamil arm (32 weeks) and the control arm (23 weeks) was more favourable than for patients with limited disease but did not achieve statistical significance.

Thus, in terms of response and survival, overall results in both treatment arms are similar, even though there were more dose reductions in the verapamil treated patients after the first course of chemotherapy. Verapamil may therefore have had some effect and it is possible that with higher plasma levels one might anticipate greater resistance modifying activity particularly as we have demonstrated a dose

**Table IV** Survival data and causes of death

| *Arm* | *Disease status* | *No. of patients* | *No. of deaths* | *Median survival* | *95% c.i. for median survival* |
|---|---|---|---|---|---|
| Verapamil | Limited | 84 | 75 | 45 weeks | 35–52 weeks |
| Control | Limited | 81 | 62 | 48 weeks | 44–56 weeks |
| Verapamil | Extensive | 27 | 25 | 32 weeks | 24–48 weeks |
| Control | Extensive | 28 | 24 | 23 weeks | 18–31 weeks |
| Verapamil | All | 111 | 100 | 41 weeks | 36–48 weeks |
| Control | All | 109 | 86 | 44 weeks | 36–49 weeks |
| Relative death rate (Verapamil/Control) | | | | = 1.17 | $P = 0.290$ |
| 95% c.i. for relative death rate | | | | = 0.87–1.57 | |

| *Causes of death* | *Verapamil* | *Control* |
|---|---|---|
| Drug toxicity | 6 | 3 |
| Tumour progression | 86 | 73 |
| Non-cancer cause (without tumour) | 0 | 1 |
| Non-cancer cause (with tumour) | 3 | 4 |
| Not known | 5 | 5 |

response effect *in vitro* (Plumb *et al.*, 1990). Such an approach is however limited by cardiovascular toxicity (Ozols *et al.*, 1987). It is known that the L-isomer of verapamil is about ten times more effective as a calcium antagonist than the D-isomer (Ferry *et al.*, 1985). However the two isomers are equipotent blockers of the fast inward current (Newrath *et al.*, 1981). Since the effects of verapamil on drug resistance do not appear to relate to calcium antagonism (Ramu *et al.*, 1984) it is conceivable that the D-isomer alone would be a more suitable agent to use clinically in view of the potential, though yet undemonstrated, reduction in cardiovascular side-effects compared to the racemic mixture. Moreover the D-stereoisomer has been shown to be equally active in terms of resistance modification in SCLC *in vitro* (Plumb *et al.*, 1990). Thus use of D-verapamil alone might be a useful approach in future clinical studies of resistance modulation. However, recent work using human tumour biopsies and cell lines now suggests that the P-glycoprotein-mediated (MDR) mechanism of resistance is unlikely to be the major factor in the drug resistance seen in small cell lung cancer (Lai *et al.*, 1989). Despite this, resistance modulation with verapamil in small cell lung cancer cell lines can still occur (Cole *et al.*, 1989), raising the possibility of an alternative mechanism of action for this particular modulating agent (Plumb *et al.*, 1989).

The failure to achieve a more substantial effect in this study may relate to the low blood levels of verapamil seen in the clinic or to the presence of other cellular mechanisms by which drug resistance develops. Further studies of resistance modulation in small cell lung cancer should perhaps employ a combination of agents, especially those where effective concentrations can be more easily achieved in the clinic. Such studies should be based on careful analysis of the mechanisms by which modulation occurs *in vitro*.

The following Consultants and Members of the West of Scotland Lung Cancer Research Group have entered patients into this study. Their contribution is gratefully acknowledged:

Dr Ron Atkinson (Belfast), Dr Steve Banham (Glasgow), Dr Alistair Dorward (Paisley), Dr John Elliot (Ayr), Dr Anna Gregor (formerly Glasgow), Dr Andy Hutcheon (Aberdeen), Dr Derek King (Aberdeen), Professor Stan Kaye (Glasgow), Dr Fergus MacBeth (Glasgow), Dr Duncan MacIntyre (Glasgow), Dr Robert Monie (Glasgow), Dr Mike Soukop (Glasgow), Dr Bryan Stack (Glasgow), Dr David Vernon (Glasgow), Dr Hosney Yosef (Glasgow).

We also gratefully acknowledge the help of the following: Miss Linda Cram (Data Manager) and Mr Jim Paul (Statistician) from the Beatson Oncology Centre, Glasgow.

This work was funded by a generous grant from the Cancer Research Campaign.

### References

ABPI DATA SHEET COMPENDIUM 1989–1990. (1990). p.1. Datapharm Publications Limited. London.

BELL, D. (1990). Written communication.

BENSON III, A.B., TRUMP, D.L., KOELLER, J.M., EGORIN, M.I., OLMAN, E.A., WITTE, R.S., DAVIS, T.E. & TORMEY, D.C. (1985). Phase I study of vinblastine and verapamil given by concurrent i.v. infusion. *Cancer Treat. Rep.*, **69**, 795–799.

COLE, S.C., FLANAGAN, R.J., JOHNSTON, A. & HOLT, D.W. (1981). Rapid high performance liquid chromatographic method for the measurement of Verapamil and Norverapamil in blood plasma or serum. *J. Chromatography*, **218**, 621–629.

COLE, S.P., DOWNES, H.F. & SLOVAK, M.L. (1989). Effect of calcium antagonists on the chemosensitivity of two multidrug-resistant human tumour cell lines which do not overexpress P-glycoprotein. *Br. J. Cancer*, **59**, 42–46.

DALTON, W.S., GROGAN, T.M., MELTZER, P.S., SCHEPER, R.J., DURIE, B.G., TAYLOR, C.W., MILLER, T.P. & SALMON, S.E. (1989). Drug-resistance in multiple myeloma and non-Hodgkins lymphoma: detection of P-glycoprotein and potential circumvention by addition of verapamil to chemotherapy. *J. Clin. Oncol.*, **7**, 415–424.

FERRY, D.R., GLOSSMANN, H. & KAUMANN, A.J. (1985). Relationship between the stereoselective negative inotropic effects of verapamil enantiomers and their binding to putative calcium channels in the heart. *Br. J. Pharmacol.*, **84**, 811–824.

FIGUEREDO, A., ARNOLD, A., GOODYAR, M., FINDLAY, B., NEVILLE, A., NORMANDEAU, R. & JONES, A. (1990). Addition of Verapamil and Tamoxifen to the initial chemotherapy of small cell lung cancer. A Phase I/II Study. *Cancer*, **65**, 1895–1902.

FINE, R.L., KOIZUMI, S., CURT, G.A. & CHABNER, B.A. (1987). Effect of calcium channel blockers on human CFU-GM with cytotoxic drugs. *J. Clin. Oncol.*, **5**, 489–495.

FOJO, A.T., UEDA, K., SLAMON, D.J., POPLACK, D.G., GOTTESMAN, M.M. & PASTAN, I. (1987). Expression of a multidrug resistance gene in human tumours and tissues. *Proc. Natl Acad. Sci. USA*, **84**, 265–269.

GOLDSTEIN, L.J., GALSKI, H., FOJO, A., WILLINGHAM, M., LAI, S.L., GAZDAR, A., PIRKER, R., GREEN, A., CRIST, W., BRODEUR, G.M., GRANT, C., LIEBER, M., COSSMAN, J., GOTTESMAN, M.M. & PASTAN, I. (1989). Expression of a multidrug resistance gene in human cancers. *J. Natl Cancer Inst.*, **81**, 116–124.

KERR, D.J., GRAHAM, J., CUMMINGS, J., MORRISON, J.G., THOMPSON, G.G., BRODIE, M.J. & KAYE, S.B. (1986). The effect of verapamil on the pharmacokinetics of adriamycin. *Cancer Chemother. Pharmacol.*, **18**, 239–242.

LAI, S.L., GOLDSTEIN, L.J., GOTTESMAN, M.M., PASTAN, I., TSAI, C.M., JOHNSTON, B.E., MULSHINE, J.L., IHDE, D.C., KAYSER, K. & GAZDAR, A.F. (1989). MDRI gene expression in lung cancer. *J. Natl Canc. Inst.*, **81**, 1144–1150.

MERRY, S., FLANIGAN, P., SCHLICK, E., FRESHNEY, R.I. & KAYE, S.B. (1989). Inherent adriamycin resistance in a murine tumour line: circumvention with verapamil and norverapamil. *Br. J. Cancer*, **59**, 895–897.

MILLER, R.L., BUKOWSKI, R.M., BUDD, G.T., PURVIS, J., WEICK, J.K., SHEPARD, K., MIDHA, K.K. & GANAPATHI, R. (1988). Clinical modulation of doxorubicin resistance by the calmodulin-inhibitor trifluoperazine: a phase I/II trial. *J. Clin. Oncol.*, **6**, 880–888.

NEWRATH, H., BLEI, I., GEGNER, A., LUDWIG, C. & ZONG, X.G. (1981). No stereoselective effects of the optical isomers of verapamil and D-600 in the heart. In *Calcium Antagonists in Cardiovascular Therapy: Experience with Verapamil*, Zanchetti, A. & Krikler, D.M. (eds) p.52–63. Excerpta Medica: Amsterdam.

OZOLS, R.F., CUNNION, R.E., KLECKER, R.W., HAMILTON, T.C., OSTCHEGA, Y., PARRILLO, J.E. & YOUNG, R.C. (1987). Verapamil and adriamycin in the treatment of drug-resistant ovarian cancer patients. *J. Clin. Oncol.*, **5**, 641–647.

PASTAN, I. & GOTTESMAN, M. (1987). Multiple drug resistance in human cancer. *New Engl. J. Med.*, **316**, 1388–1393.

PLUMB, J.A., MILROY, R. & KAYE, S.B. (1989). Is there a role for resistance modifiers in cancer chemotherapy? *Br. J. Cancer*, **60**, 473 (abstr).

PLUMB, J.A., MILROY, R. & KAYE, S.B. (1990). The activity of Verapamil as a resistance modifier *in vitro* in drug resistant human tumour cell lines is not stereospecific. *Biochem. Pharmacol.*, **39**, 787–792.

PRESANT, C.A., KENNEDY, P.S., WISEMAN, C., GALA, K., BOUZAGLOU, A., WYRES, M. & NAESSIG, V. (1986). Verapamil reversal of clinical doxorubicin resistance in human cancer. A Wiltshire Oncology Medical Group pilot Phase I–II study. *Am. J. Clin. Oncol.*, **9**, 355–357.

RAMU, A., SPANIER, R., RAHAMINOFF, H. & FUKS, Z. (1984). Restoration of doxorubicin responsiveness in doxorubicin-resistant P388 murine leukaemia cells. *Br. J. Cancer*, **50**, 501–507.

SOUHAMI, R.L., BRADBURY, I., GEDDES, D.M., SPIRO, S.G., HARPER, P.G. & TOBIAS, J.S. (1985). Prognostic significance of laboratory parameters measured at diagnosis in small cell carcinomas of the lung. *Cancer Res.*, **45**, 2878–2882.

SUGAWARA, I., KATAOKA, I., MORISHITA, Y., HAMADA, T., TSURUO, T., ITOYAMA, S. & MORI, S. (1988). Tissue distribution of P-glycoprotein encoded by a multidrug-resistant gene as revealed by a monoclonal antibody, MRK16. *Cancer Res.*, **48**, 1926–1929.

THIEBAULT, F., TSURUO, T., HAMADA, H., GOTTESMAN, M.M., PASTAN, I. & WILLINGHAM, M.C. (1987). Cellular localization of the multidrug-resistance gene product P-glycoprotein in normal human tissues. *Proc. Natl. Acad. Sci. USA*, **84**, 7735–7738.

TSURUO, T., IIDA, H., TSUKAGOSHI, S. & SAKURAI, Y. (1982). Increased accumulation of vincristine and adriamycin in drug-resistant P388 tumour cells following incubation with calcium antagonists and calmodulin inhibitors. *Cancer Res.*, **42**, 4730–4733.

YALOWICH, J.C., ZUCALI, J.R., GROSS, M. & ROSS, W.E. (1985). Effects of Verapamil on etoposide, vincristine and adriamycin activity in normal human bone-marrow granulocyte-macrophage progenitors and in human K562 leukaemia cells *in vitro*. *Cancer Res.*, **45**, 4921–4924.